# Daylight photodynamic therapy for Bowen's disease^☆^^☆☆^

**DOI:** 10.1016/j.abd.2019.11.012

**Published:** 2020-05-11

**Authors:** Carla Corrêa Martins, Renato Marchiori Bakos, Manuela Martins Costa

**Affiliations:** Department of Dermatology, Hospital de Clínicas de Porto Alegre, Porto Alegre, RS, Brazil

Dear Editor,

Bowen's disease (BD) is considered an indolent neoplasm with high cure rates after non-surgical treatments, which are often the first-line therapy.^1^

The effectiveness of daylight photodynamic therapy (dPDT) in skin tumors has been demonstrated in studies with basal cell carcinomas.^2^ Moreover, two case reports of BD treatment with dPDT showed complete response in three BD lesions.^3^, ^4^ To date, no prospective studies have attempted to assess dPDT efficacy in BD treatment. The aim of this clinical trial was to assess the efficacy of dPTD for BD lesions at three months of follow-up.

A prospective study was conducted with consecutively selected samples among patients with histological diagnosis of BD. The treatment was performed between May 2016 and May 2017, in all seasons of the year.

The session followed the photographic registration protocol prior to treatment, with a camera (Canon® Powershot SX 500 IS) and a digital dermoscope (Fotofinder®). A sunscreen with organic filter (SPF 30 ) was applied on sun-exposed skin areas. Fifteen minutes later, the lesion surface was prepared by gentle removal of scales and scabs by curettage. A thin layer of 16% MAL cream was applied to the lesion. Patients started the daylight exposure within 30 min of the photosensitizer precursor application, and the exposure continued for two hours in an area within the hospital grounds.

Patients received one cycle of dPDT consisting of two treatment sessions, one week apart. The weather conditions, temperature, and maximum daily ultraviolet index (UVI) for Porto Alegre (Brazil) were obtained from the website of the Center for Weather Forecasting and Climate Studies.^5^

Treatment efficacy was assessed three months after dPDT. The primary endpoint was complete response, determined by clinical evaluation. Lesion responses were classified as complete, partial response (< 25%, 25–75%, or >75% response), or no response. The clinical assessment, conducted by a dermatologist, considered the presence of persistent erythema, hyperkeratotic areas, reduction of lesion size, and other dermoscopic data such as the occurrence of typical dotted or glomerular vessels.

The pain score was recorded each 30 min during the daylight exposure period and weekly following the treatment, by telephone questionnaire. It was scored using a visual analog scale ranging from 0 to 10 (0 = no pain, 10 = worst pain ever felt). In the evaluations, the main signs and symptoms were queried.

Nineteen patients, with 24 BD lesions, were included in the study (Table 1). Ten lesions were located on the lower limbs, five on the head/neck area, five on the trunk, and four on the upper limbs. The majority of the lesions received the dPDT sessions between 10 a.m. and 2 p.m. (*n* = 16; 66.7%), on sunny or partially cloudy days (*n* = 15; 62.5%). The mean temperature was 19.91 °C (± 5.2) at the first session and 17.54 °C (± 4.6) at the second session. The mean ultraviolet index (UVI) was 3.25 (± 2.8).

**Table 1:** Demographic characteristics of patients.

Total (*n* = 19)	
*Age, mean (± SD)*	69.7 years (± 10.6 years)

*Gender, n (%)*	
Male	12 (63.2%)
Female	7 (36.8%)


*Transplanted, n (%)*	
Yes	6 (31.6%)
No	13 (68.4%)

*Comorbidities, n (%)*	
Yes	15 (79.0%)
No	4 (21.0%)

*Phototype, n (%)*	
II	16 (84.2%)
III	2 (10.5%)
IV	1 (5.3%)

Three months after dPDT, six lesions (25%) showed a complete clinical response, eight (33%) lesions showed > 75% partial response, four (8%) presented 25–75% partial response, two (8%) showed < 25% partial response, and four (8%) presented no response.

Lesions that presented a better response were located on the sun-exposed area, like the upper limbs and head/neck area (*p* = 0.01); however, no specific group of patients benefiting from the technique was observed (Table 2).

**Table 2:** Comparison between good responders ( clinical response > 75% or complete) and less responsive lesions with regard to clinical and climatic data after three months of follow-up.

	Response^a^		*p*-Value*
	<75%	≥75%	
	(*n* = 10)	(*n* = 14)	
*Sex, n (%)*			0.73
Female	5 (45.5)	6 (54.5)	
Male	5 (38.5)	8 (61.5)	

*Age, years, mean (± SD)*	70.6 (± 2.8)	67.1 (± 2.7)	0.39
*Lesion size, mm, mean (± SD)*	19.7 (± 8.1)	21.0 (± 15.8)	0.87

*Transplant recipient, n (%)*			0.89
Yes	4 (40.0)	6 (60.0)	
No	6 (42.9)	8 (57.1)	

*Comorbidities, n (%)*			0.93
Yes	8 (42.1)	1 (57.9)	
No	2 (40.0)	3 (60.0)	

*Climatic condition, n (%)*			0.83
Sunny and/or partially cloudy on both applications	6 (40.0)	9 (60.0)	
Other combined conditions involving cloudy or rainy days	4 (44.4)	5 (55.6)	

*UVI, mean (± SD)*	2.8 (± 2.8)	3.6 (± 2.8)	0.43

*Skin type, n (%)*			0.24
II	9 (42.9)	12 (57.1)	
III	0.00	2 (100.0)	
IV	1 (100.0)	0.00	

*Body site, n (%)*			0.01
Head/neck	1 (20.0)	4 (80.0)	
Trunk	5 (100.0)	0	
Lower limbs	4 (40.0)	6 (60.0)	
Upper limbs	0	4 (100.0)	

a Chi-squared test for sex, transplant recipients, climatic condition, phototype, and body location variables; Student's *t*-test for age and lesion size variables; Mann–Whitney *U*-test for UVI variable.

* *p* ≤ 0.05.

The pain score perception during treatment was 0 in 79.2% of the treated lesions (*n* = 19), with a median visual pain score of 0 (range 0–3) in both sessions. The adverse effects most frequently reported by the patients were scaling and redness.

The findings of this study suggest that dPDT is a feasible alternative treatment for BD in selected cases. Complete response was found in six lesions after three months of follow-up, demonstrating that some cases would benefit from dPDT. Moreover, in 14 (58.3%) lesions improvement was observed in more than 75% of the lesion area, supporting its use as a neoadjuvant alternative. However, these findings for dPDT show that, when compared to cPDT, there were fewer complete response cases, demonstrating that the latter might still be generally preferable for BD patients.^1^

Sample size and lack of a control group were limitations of the study, as well as the follow-up time. Future studies evaluating efficacy in a longer follow-up may corroborate the findings of this study. It is also assumed that re-treatment of lesions that have partially improved may increase the proportion of lesions with complete response over a long-term follow-up.

## Financial support

Research and Events Incentive Fund of Hospital de Clínicas de Porto Alegre.

## Authors’ contributions

Carla Corrêa Martins: Statistical analysis; conception and planning of the study; drafting and editing of the manuscript; collection, analysis, and interpretation of data; participation in design of the study; intellectual participation in the propaedeutic and/or therapeutic conduct of the studied cases; critical review of the literature; critical review of the manuscript.

Renato Marchiori Bakos: Approval of the final version of the manuscript; conception and planning of the study; drafting and editing of the manuscript; collection, analysis, and interpretation of data; participation in design of the study; intellectual participation in the propaedeutic and/or therapeutic conduct of the studied cases; critical review of the manuscript.

Manuela Martins Costa: Conception and planning of the study; drafting and editing of the manuscript; participation in design of the study.

## Conflicts of interest

None declared.

## References

[1] Morton C.A., Birnie A.J., Eedy D.J. (2014). British Association of Dermatologists’ guidelines for the management of squamous cell carcinoma in situ (Bowen's disease) 2014. Br J Dermatol.

[2] Wiegell S.R., Skødt V., Wulf H.C. (2014). Daylight-mediated photodynamic therapy of basal cell carcinomas - an explorative study. J Eur Acad Dermatology Venereol.

[3] Pérez-Pérez L., García-Gavín J. (2014). Terapia fotodinámica con luz de día en la enfermedad de Bowen. Piel.

[4] Safar R., Alkhars A., Tallegas M., Korsaga-Some N., Machet L. (2019). Successful treatment for extensive Bowen's disease using daylight-mediated photodynamic therapy. Acta Derm Venereol.

[5] INPE – Instituto Nacional de Pesquisas Espaciais [Internet]. Porto Alegre: Centro de Previsão de Tempo e Estudos Climáticos. Available from: http://www.cptec.inpe.br/ [accessed 26.05.17].

